# Study on Low-Velocity Impact and Residual Compressive Mechanical Properties of Carbon Fiber–Epoxy Resin Composites

**DOI:** 10.3390/ma17153766

**Published:** 2024-07-31

**Authors:** Xueyuan Qiang, Te Wang, Hua Xue, Jun Ding, Chengji Deng

**Affiliations:** 1 National Center for Composites Quality Testing & Inspectionl (Hubei), Hubei Zery Testing Technology Co., Ltd., Xiangyang 441000, China; 2 The State Key Laboratory of Refractories and Metallurgy, Wuhan University of Science and Technology, Wuhan 430081, China

**Keywords:** CF/EP composites, low-velocity impact, impact-damage mechanism, compression after impact, failure mechanism

## Abstract

Room temperature drop hammer impact and compression after impact (CAI) experiments were conducted on carbon fiber–epoxy resin (CF/EP) composites to investigate the variation in impact load and absorbed energy, as well as to determine the residual compressive strength of CF/EP composites following impact damage. Industrial CT scanning was employed to observe the damage morphology after both impact and compression, aiding in the study of impact-damage and compression-failure mechanisms. The results indicate that, under the impact load, the surface of a CF/EP composite exhibits evident cratering as the impact energy increases, while cracks form along the length direction on the back surface. The residual compressive strength exhibits an inverse relationship with the impact energy. Impact damage occurring at an energy lower than 45 J results in end crushing during the compression of CF/EP composites, whereas energy exceeding 45 J leads to the formation of long cracks spanning the entire width of the specimen, primarily distributed symmetrically along the center of the specimen.

## 1. Introduction

Resin matrix composites have broad development prospects due to their brilliant fatigue resistance, corrosion resistance, and shock absorption. In addition, resin matrix composites possess the advantages of a simple forming process, strong plasticity of the material structure, and performance, and are widely used in the automotive industry, aerospace industry, and coal-mining industry [1,2,3,4]. Carbon-fiber-reinforced composites, a pivotal category within resin matrix composites, find extensive utilization in the aerospace sector, shipbuilding, and other fields owing to their higher strength, lower density, and better fatigue resistance [5,6,7,8,9,10]. However, with the continuous improvement of material performance requirements, particularly when confronted with dynamic impact loads (for example, the effect of bird flocks, hail, and other foreign objects on aircraft during flight), the research on the impact resistance and damage mechanism of carbon-fiber-reinforced plastics (CFRP) has become crucial [11,12,13,14,15,16,17].

In practical applications, carbon-fiber composites may face a variety of impact loads from collisions, impacts, and accidents. Therefore, many scholars have implemented studies on their impact resistance and damage mechanism [18,19,20,21]. Liu et al. [22] prepared hybrid composite laminates composed of carbon-fiber unidirectional layers and braided layers by the resin-impregnation method and carried out drop-weight impact experiments on different laminated samples. The results show that the laminated samples affect the impact response of the composite laminates by changing the overall bending stiffness. Kumar et al. [23] prepared SiC/Al composites and explored the influence of SiC content on its shock resistance and wear resistance. The results indicate that the impact strength and specific wear of the composites with SiC volume fraction is 9%. Some studies have already proven that glass fiber possesses strong impact resistance. Therefore, to further enhance the properties of CFRP in low-velocity impact tests, some researchers contemplated adding a glass-fiber layer to it [24,25,26,27]. Lei et al. [28] investigated the low-velocity impact and post-compression behavior following the impact of various fiber composites and flame-retardant epoxy resin. The findings prove that introducing glass fiber can change the impact-damage mode of laminates and effectively improve the impact resistance and compressive impact strength of laminates. When it comes to the study of the damage behavior of carbon-fiber-reinforced resin matrix composites, in addition to routine performance testing and cross-sectional morphology analysis, researchers can also utilize finite-element methods to simulate the behavior of carbon-fiber composites under impact loads and predict their damage mechanisms [29,30,31]. Researchers [32,33,34,35,36,37,38] have carried out research on the mechanical properties of materials with single/mixed reinforcements for low-velocity impact or/and post-impact damage and analyzed the material failure mechanisms.

In summary, researching the impact resistance and damage mechanism of CFRP is not only of great significance for improving the performance and reliability of materials but also provides theoretical guidance and technical support for its widespread applications in aviation, automotive, and other fields [39,40,41]. Most studies adopt ultrasonic and ray damage research means and fewer of them use the industrial CT method. The existing literature on T700 carbon-fiber-reinforced epoxy-resin matrix composites’ low-speed impact and post-impact damage research appears to be insufficient, while the utilization of industrial CT to study the impact damage is little reported. The existing research mainly focuses on the influence of reinforcing materials and preparation methods on the properties of composites, but the damage mechanisms of composites under varying loads may turn out to be corresponding changing forms. Therefore, this study used T700 carbon fiber as the reinforcing material, QC350 epoxy resin was used as the matrix, and composites were prepared by the vacuum introduction molding method. Through low-speed impact and post-impact compression tests, based on the non-destructive characterization method of industrial CT, the damage failure mechanism of laminated plates was analyzed, and the effect of impact energy on residual compressive strength was investigated.

## 2. Materials and Methods

### 2.1. Materials

The reinforcement material is T700 carbon-fiber multidirectional cloth (Toray, Tokyo, Japan), the matrix material is epoxy resin QC350, and the ratio of QC350 is QC350A:QC350B = 100:30. The plywood layup is [(45/−45)/(0/90)]. The nominal thickness of the single layer is 0.29 mm, and the thickness of the specimen is 5.7 mm. The performance parameters of T700 carbon-fiber multidirectional cloth are detailed in Table 1, and the performance parameters of epoxy resin QC350 are detailed in Table 2.

**Figure 1:**
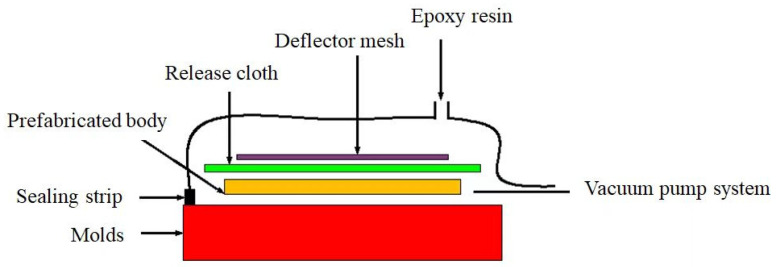
Schematic diagram of vacuum infusion molding.

CF/EP composites were prepared by the vacuum introduction molding method, and the preparation schematic is shown in Figure 1. In accordance with the prescribed lay-up order, the prefabricated body was laid flat to the mold surface, and the auxiliary materials, such as the infusion net and the inlet–outlet hose, were laid in order to strengthen the flow performance of the epoxy resin. A vacuum-bag film with a length and width of 400 mm larger than the sample was laid flat, and the mold cavity was sealed with sealing tape. At room temperature, the vacuum system was used to achieve the pressure of 0.1 Pa between the vacuum bag and the mold cavity, and the pressure was maintained for 1 h. With the assistance of the atmospheric pressure effect, the epoxy resin slowly entered the cavity along the infusion network until the resin filled up the entire mold and impregnated the preformed body, and then it was cured at 80 °C.

### 2.2. Testing and Characterization

**Figure 2:**
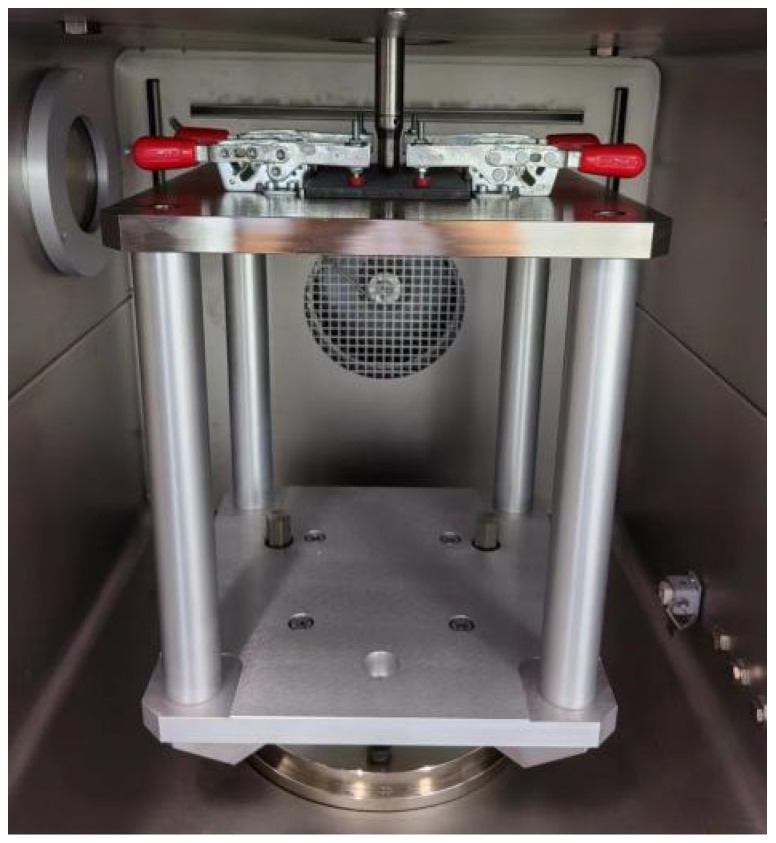
Clamping diagram of low-velocity impact test specimen.

According to ASTM D7136/D7136M-2020 [42], the low-velocity impact test was carried out by using a φ16 mm hemispherical punch with a weight of 4.3 kg, and the weight of the whole drop-weight system was 5.482 kg (the equipment model and manufacturer are CEAST 9350, Instron, Pianezza, Italy). The sample should be placed on the impact fixed base, and the position of the sample should be adjusted appropriately. In that case, the end of the punch could accurately impact the center of the sample. Put down four plastic calipers in turn (as shown in Figure 2) to ensure that the calipers can firmly fix the sample, and the clamping force on the sample is basically the same so as to prevent the sample from slipping during the drop hammer impact process, affecting the accuracy of the test results. According to the test requirements under different amounts of energy, the test parameters were set in turn. The impact energies were sequentially administered at 25 J, 35 J, 45 J, 65 J, 85 J, 105 J, and 125 J, resulting in impact velocities of 3.02 m/s, 3.57 m/s, 4.05 m/s, 4.87 m/s, 5.57 m/s, 6.19 m/s, and 6.75 m/s, respectively. At the same time, the drop hammer impact test in turn should be carried out using the anti-secondary impact device. Through the data acquisition system, the key performance indexes, such as energy absorption and bearing load of the sample during the impact process, are obtained, and the impact resistance of the material is evaluated.

According to the ASTM D7136 standard, the velocity $v\left(t\right)$, displacement $\delta \left(t\right)$ and absorbed energy $E_{a}\left(t\right)$ of the punch varying with time can be obtained and are shown in Equation (1), Equation (2), and Equation (3), respectively.

(1) $$v\left(t\right)=v_{i}+gt-\int_{0}^{t}\frac{F\left(t\right)}{m}dt$$

where $v\left(t\right)$ is the velocity of the punch at moment t in m/s. $v_{i}$ is the initial velocity of the punch in m/s. g is the acceleration of gravity in m/s^2^.$F\left(t\right)$ is the contact force at moment t in N. m is the mass of the punch in kg.

(2) $$\delta_{t}=\delta_{i}+v_{i}t+\frac{gt^{2}}{2}-\int_{0}^{t}\left(\int_{0}^{t}\frac{F\left(t\right)}{m}dt\right)dt$$

where $\delta \left(t\right)$ is the punch displacement in mm. $\delta_{i}$ is the starting displacement of the punch at t = 0 in mm.

(3) $$E_{a}\left(t\right)=\frac{m\left({v_{i}}^{2}-v{\left(t\right)}^{2}\right)}{2}+mg\delta \left(t\right)$$

where $E_{a}\left(t\right)$ is the absorbed energy at time t in J.

The compressive damage load of the plywood obtained from the compression test can be calculated from Equation (4) to obtain the compressive strength $F^{CAI}$.

(4) $$F^{CAI}=P_{max}/A$$

where $F^{CAI}$ is the compressive strength in MPa. $P_{max}$ is the maximum load in N at the time of specimen destruction. A is the cross-sectional area of the specimen in mm^2^.

**Figure 3:**
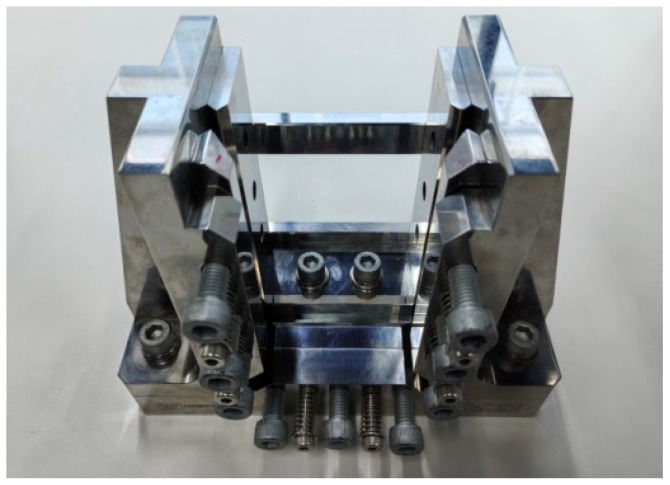
Schematic diagram of the remaining compression experimental fixture after impact.

The residual compression test under different impact energies was carried out by an electronic universal testing machine (Instron 5989, Instron; the physical picture of the device is shown in Figure 3). The sample after the impact test was clamped to the compression fixture to ensure that the sample and the fixture were aligned, and the damage position of the sample emerged at the center of the fixture. At the same time, the fixture was equipped with an anti-bending device to prevent the introduction of bending loads other than axial compression during the compression process. The fixture specimen was positioned within the pressure plates of the testing apparatus and subjected to a compression load applied longitudinally along its axis at a rate of 1.25 mm/min until reaching its peak, where the load decreased by 30%. The apparent morphology of the test sample was observed by industrial CT.

## 3. Results and Discussion

### 3.1. Low-Speed Impact Experiment

**Figure 4:**
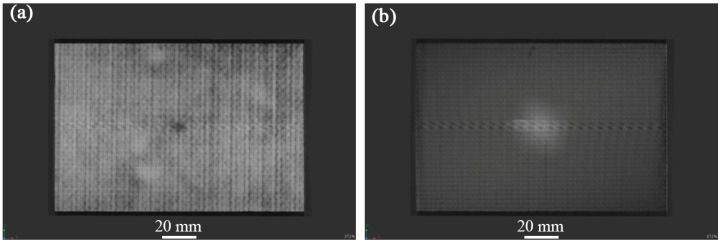
CT damage atlas of sample after 45 J impact energy (**a**) specimen face, (**b**)specimen back.

**Figure 5:**
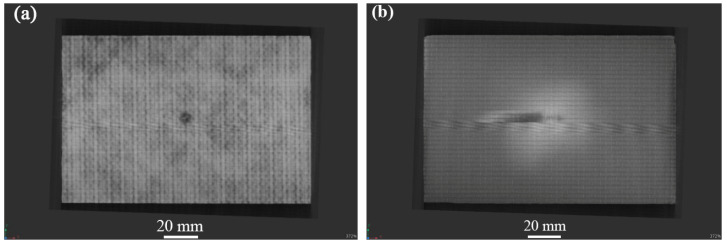
CT damage atlas of sample after 105 J impact energy (**a**) specimen face, (**b**)specimen back.

**Figure 6:**
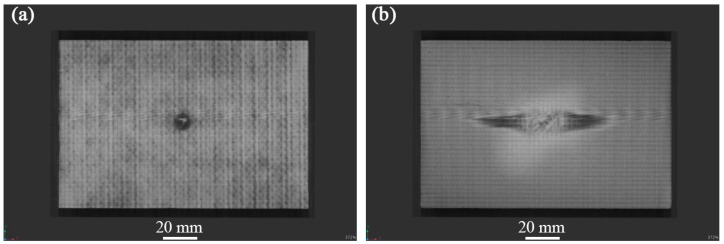
CT damage atlas of sample after 125 J impact energy (**a**) specimen face, (**b**)specimen back.

Figure 4, Figure 5 and Figure 6 show the non-destructive CT inspection patterns of CF/EP composites on the front and back of the specimens after impacts at different energies. It can be seen that round craters of different sizes and depths are produced on the impact surface, and long cracks along the axial direction appear on the back surface. It can be observed from Figure 4 that, when the impact energy is 45 J, while there are only shallow pits on the surface of the sample, there are only a few short cracks and inconspicuous damage. At an impact energy of 105 J, an increasing incidence of extensive internal cracks becomes discernible within the specimen (Figure 5). Figure 6 shows the surface and back of the specimen after the 125 J impact energy impact test, from which the back of the specimen is affected by tensile stress can be seen, resulting in fiber fracture, but the damage does not penetrate the entire specimen. It can be acknowledged that, with the increase of impact energy, the longer the crack, the greater the impact damage will be [43,44,45].

**Figure 7:**
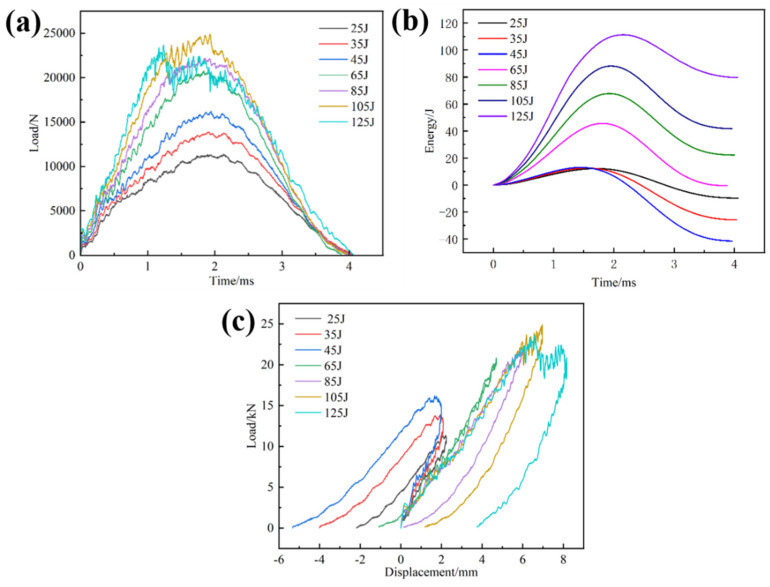
Load/energy-time curves and load-displacement curves of specimens under different impact energy levels; (**a**) load-time curves, (**b**) energy-time curves, (**c**) load-displacement curves.

Figure 7 demonstrates the load-time curve, energy-time curve, and load-displacement curve of CF/EP composites under different energy impacts. It can be observed from Figure 7a that the curves display three stages: linear loading, platform, and nonlinear unloading. Under low-energy impact, the sample undergoes a short platform stage and then enters the unloading stage, indicating that there is a debonding of the sample matrix from the fibers and a small amount of fiber breakage. Because of the rapid impact process, in a very short period of time, when the phenomenon of debonding and fiber breakage is obviously intensified, there is an outstanding downward trend at the curve until the specimen is damaged. The composite’s failure modifies from matrix failure to fiber fracture, and the fiber is capable of resisting the punch load. Therefore, when the energy exceeds 45 J, the load increases rapidly, which is the “critical value” of the composite-failure mode transformation. With the energy reaching 125 J, the load value of the platform stage fluctuates significantly, indicating that a wide area of fiber and matrix failure becomes evident on the back of the sample [46,47]. When the integrity of the laminated plate structure is destroyed, its bearing capacity experiences a corresponding decrease, and the impact force holds a downward trend in the second half of the platform stage.

It is evident from Figure 7b that the absorbed energy (dissipated energy) in the composite sample is less than the initial impact energy. This is owing to the damage, such as fiber breakage, in the sample consuming part of the energy. When the energy increases to 45 J, the peak absorption energy undergoes a leapfrog increase, and the primary mechanism of energy dissipation transforms from matrix cracking to fiber fracture progressively, which is consistent with the conclusion of Figure 7a. With the increasing impact energy, the composites will absorb more energy.

It can be indicated from Figure 7c that the whole load-displacement curve shows two stages, namely the linear loading stage and the nonlinear unloading stage. However, at low energy impacts, three phases are presented, which are the linear loading phase, the nonlinear loading phase, and the nonlinear unloading phase, which are more obvious. During the loading stage, the curve is linear and accompanied by a small jagged fluctuation. It shows that, during the impact-loading process, the composite laminate contains a large number of staggered carbon fibers to continuously resist the pressure applied by the punch, resulting in a continuous reciprocating ‘pressure-resistance’ phenomenon, and the stiffness change is small or even ignored. During the loading process, the majority of the punch’s kinetic energy is stored as elastic strain energy. As the punch applies sustained pressure to the laminate, the composite experiences matrix failure accompanied by progressive fiber breakage until the load reaches its peak capacity. When the impact force reaches the peak, the punch rebounds under the action of accumulated elastic strain energy, and the load-displacement curve also enters the nonlinear unloading stage. When the load is unloaded to zero, the displacement on the curve does not return to the initial position, which is caused by the impact-induced irreversible damage of the laminate and a small part of the energy dissipation loss [37,48,49].

### 3.2. Compression Experiment after Impact

**Figure 8:**
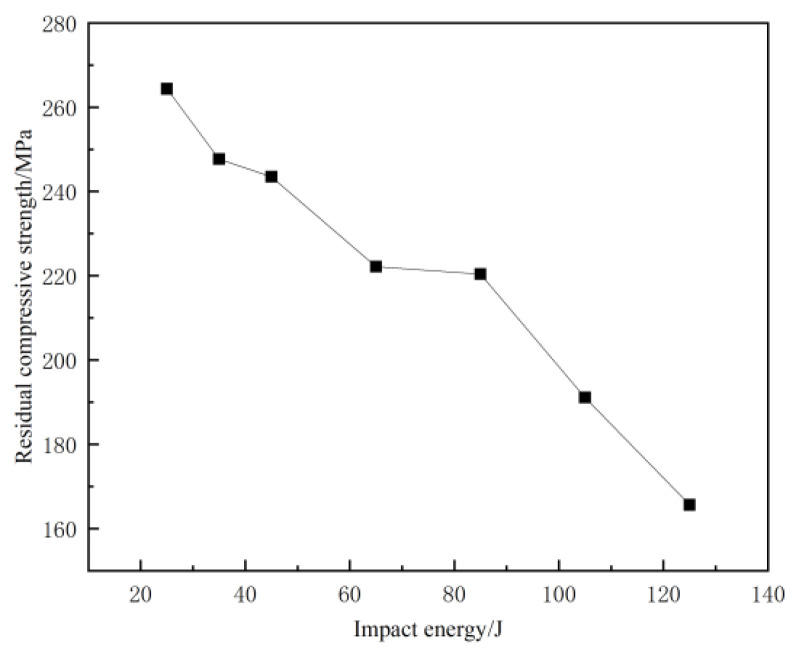
Residual compressive strength curves at different impact energies.

The residual compressive strength and damage that form the CF/EP composites under different impact energies at room temperature are listed in Table 3. It can be seen from Table 3 that the damage mode of the sample is end crushing, while the impact energy is less than 45 J. Compared to Figure 4, it can be observed that, after the sample is impacted by low energy, the impact surface forms pits of different depths, and micro-damage, such as surface matrix cracking, occurs. The impact-damage area is small, and the residual bearing capacity of the sample is sufficient. When the impact energy exceeds 45 J, the damage mode of the specimen changes to LDM (lateral multi-layered expansion) damage, and the residual compressive bearing capacity of the specimen is significantly reduced or even fails, which is consistent with the findings in the previous section. The residual compressive strength at the maximum energy of 125 J is 165.62 MPa, which is 37% lower than that at 25 J. According to the content of Table 3, the curve of impact energy surplus compressive strength shown in Figure 8 is drawn. Figure 8 indicates that, with increasing impact energy, the residual compressive strength of the sample decreases gradually, and the residual bearing capacity of the sample decreases progressively.

**Figure 9:**
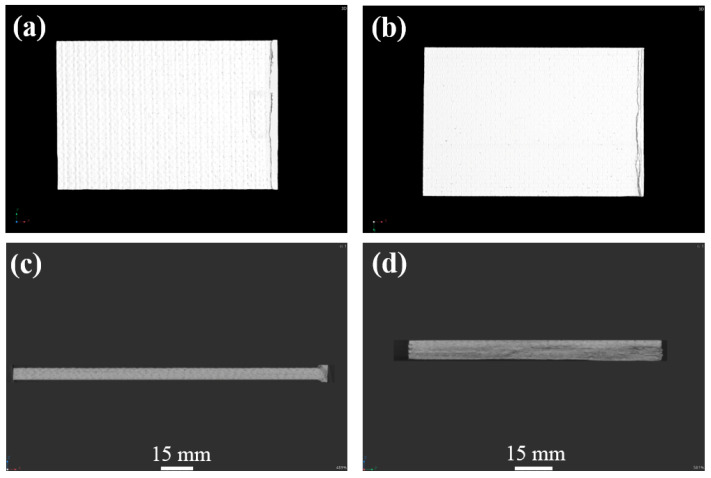
CT atlas of compressive test specimen after 35 J impact energy; (**a**) specimen face, (**b**) specimen back, (**c**) specimen length direction end, (**d**) specimen width direction end.

Figure 9 illustrates the damage morphology of the CF/EP composites compressed after 35 J energy impact. The specimen shows obvious end-crushing damage. The specimen is subjected to compressive loading, and the compression surface shows delamination and cracks of different lengths, and even narrow delamination pores. The front and back surfaces showed flat cracks extending along the entire width direction, and the rest of the plane showed unnoticeable visual compression damage. At the end of the specimen, along the thickness of the long side, there is a compression damage of about 90°, and the fibers show destructive fracture. Because the end of the specimen is subjected to a concentrated compression load, the adhesive strength between the end fibers and resin decreases, and debonding delamination damage easily occurs. With the further increase of the load, the specimen end is subject to concentrated contact stress. The load is insufficient to transfer through the specimen end to the impact of the small damaged area. The end of the damage is further aggravated. The fiber cannot effectively carry the compression load, resulting in the end of the fiber fracture, and the matrix cracks and delaminates. The specimen at the end of the compression collapses.

**Figure 10:**
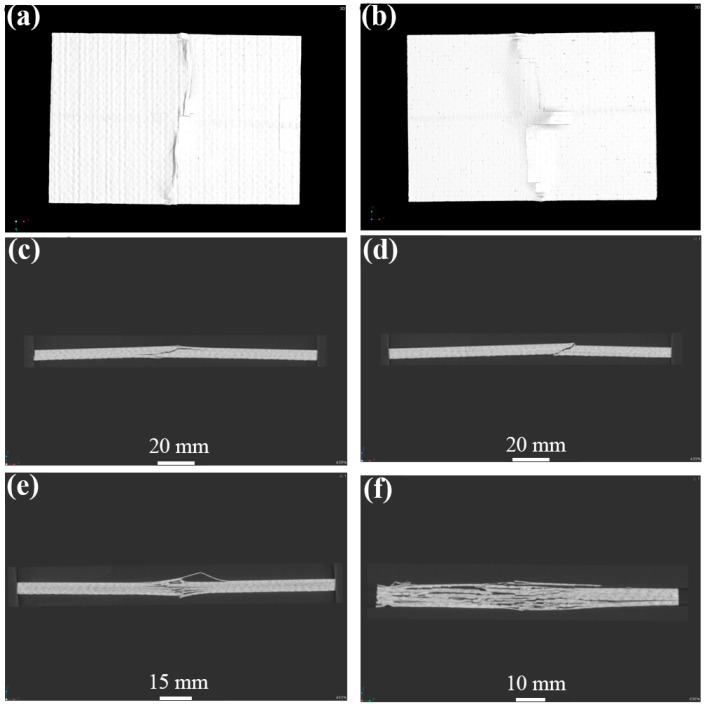
CT atlas of compressive test specimen after 45 J impact energy; (**a**) specimen face, (**b**) specimen back, (**c**,**d**) specimen length direction side, (**e**) specimen center of length direction, (**f**) specimen center of width direction.

**Figure 11:**
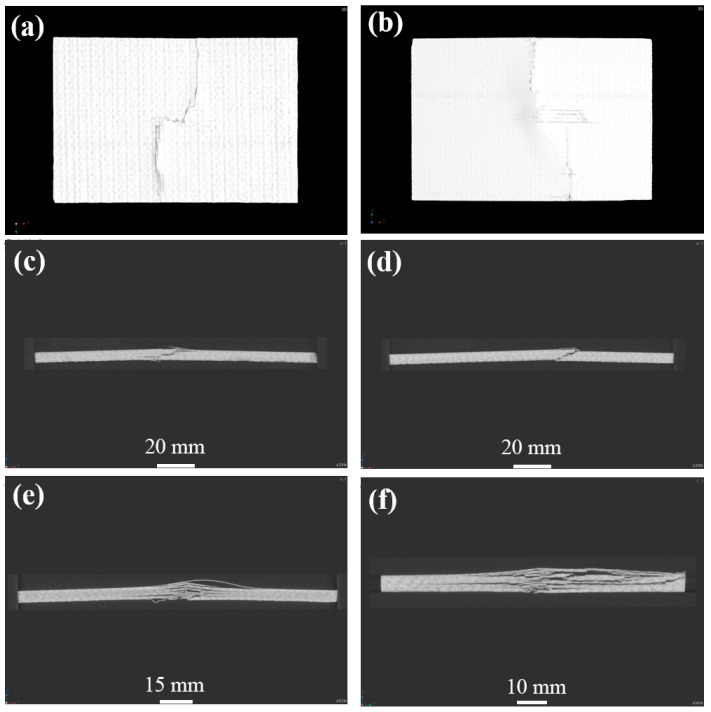
CT atlas of compressive test specimen after 105 J impact energy; (**a**) specimen face, (**b**) specimen back, (**c**,**d**) specimen length direction side, (**e**) specimen center of length direction, (**f**) specimen center of width direction.

**Figure 12:**
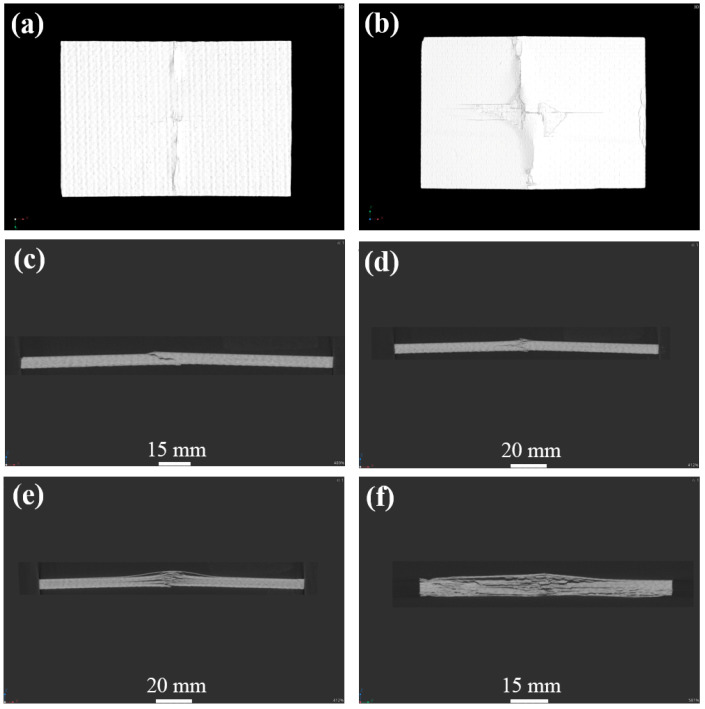
CT atlas of compressive test specimen after 125 J impact energy; (**a**) specimen face, (**b**) specimen back, (**c**,**d**) specimen length direction side, (**e**) specimen center of length direction, (**f**) specimen center of width direction.

Figure 10, Figure 11 and Figure 12 display the damage picture of compression specimens of CF/EP composites after 45J, 105 J, and 125 J energy impacts. Under the axial compression load, matrix cracks first appeared in the center part of the impact surface, accompanied by a little delamination and fiber pullout. On the front side, long cracks across the entire width were common, with surface fiber debonding and delamination, and the surface matrix appeared to be wrinkled and raised. Compression damage on the back of the specimen is more prominent, such as long cracks, fiber breakage, debonding delamination, and other injuries, with transverse and longitudinal fiber partially pulled out and fractured, and the specimen is severely buckled, with even the formation of “arch bridge” delamination as well. There are cracks along the transverse expansion, with the formation of long cracks across the width of the specimen, and a basically symmetrical distribution along the center of the specimen but, in the axial direction, only held a small distance, resulting in catastrophic damage failure. From the thickness direction, clear and obvious intralaminar cracks and delaminations appeared inside the specimen, and there was an inclined shear fracture of about 45° spanning almost the entire thickness direction. Even a long strip of porosity and compression collapse were also present. Deeper observation of the internal damage at the location of the impact point of the specimen proves that the damage is even more severe. Observation from the long edge direction shows that the center of the long edge of the specimen shows significant delamination, fiber breakage, and other destructive damage, and the back of the specimen is raised. The findings from the wide edge direction reveal a heightened incidence of debonding and delamination characterized by varying degrees of delamination severity, forming delamination damage of various lengths and sizes, as well as oblique cracks and flat cracks, and the oblique cracks will penetrate through the multilayered fibers and resins to form shear-type damage. Because of under-compressive loading, the stress formed at the end of the specimen is continuously transferred to the vicinity of the impact defect. The stress is redistributed and concentrated, making cracks and delamination in the middle of the damaged region expand significantly and form a penetrating fracture, which is the specimen’s serious failure.

**Figure 13:**
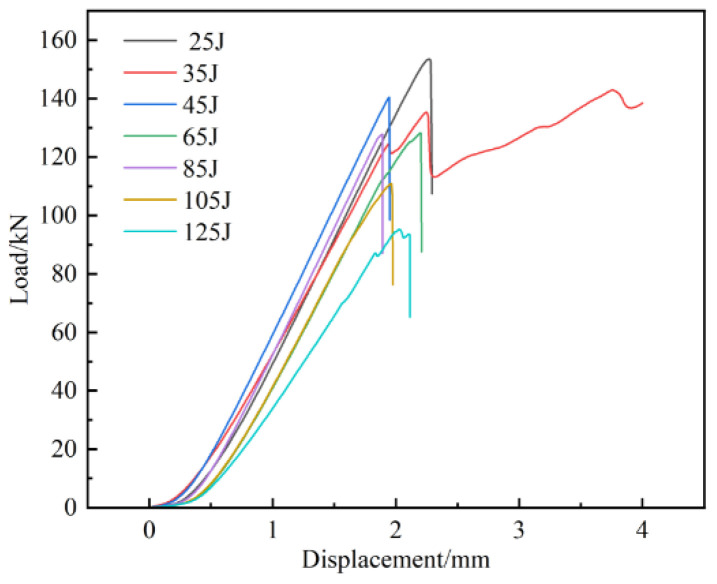
Residual compressive load-displacement curves of CF/EP composites.

As shown in Figure 13, the overall CAI load-displacement curves at different energies show a linear relationship. In the initial stage, with the onset loading of the load, the displacement of the indenter into the end of the specimen increases slowly and has a nonlinear relationship. With the further increase in the compression load, the load-displacement curve appears to be obviously linear, and finally, the load is presented as falling off a cliff. At this time, the specimen with an initial impact damage has reached the load-bearing limit, and the specimen appears to have clearly visible fiber pullout, delamination, matrix cracking, and other serious damage and has been destroyed. Through observation, it can be seen that sudden brittle fracture occurs in the specimens when the impact energy is below 45 J. The compression load reaches its limit and then suddenly decreases, which is due to the smaller impact energy, lighter damage, and the need to withstand more load on the fiber and matrix under the compression load [50,51]. Before the damage occurs at the interface, the load-carrying capacity of the specimen has reached its limit and thus brittle fracture occurs. When the specimen is above 45 J, because the specimen failed to appear along the width direction of the complete fracture, the specimen has produced localized damage, and the surface layer of fibers has been visually fractured or pulled out and cannot effectively carry any more compressive load. So, the bearing capacity is reduced, and the matrix has become the main bearer of the compressive load to strengthen the generation of irreversible plastic deformation [52].

## 4. Conclusions

(1) After the CF/EP composites were impacted by low speed, the specimens would have pits on the front side. The pits will be deeper with the increase of impact energy. In addition, in terms of the back side of the matrix-cracking phenomenon, the greater the energy is, the more significant the back damage will be;

(2) Impact damage has a great influence on the residual compressive strength of CF/EP composites. The residual compressive strength decreases with the increase of impact energy. The critical energy value is 45 J, and the end crushing occurs after compression of the sample below 45 J. The delamination propagation (LDM) damage occurs from the middle to the lateral side of the sample after compression of the sample above 45 J, and the residual compressive strength decreases sharply when the impact energy is over 45 J. The residual compressive strength at the lowest energy of 25 J is 264.37 MPa, and the residual compressive strength at the highest energy of 125 J is 165.62 MPa, which is 37% lower than the residual compressive strength at 25 J;

(3) After the CAI test, the impact surface of CF/EP composites produced cracks along the length and width directions, and the cracks gradually expanded transversely to the edge of the sample from the end of the impact cracks as the impact energy increased.

## Figures and Tables

**Table 1:** Property parameters of T700 carbon fibers.

Tensile Strength/MPa	Young’s Modulus/MPa	Elongation/%	Fineness/(g/km)	Density/(g·cm^−3^)	Fiber Volume Fraction/%	Thickness/mm	Diameter/μm
4900	2300	2.1	800	1.80	54.5	0.42	7

**Table 2:** Property parameters of QC350 epoxy resin.

Mixed Viscosity/(CPS)	Glass-Transition Temperature/°C	Tensile Strength/MPa	Modulus/MPa
250~300	75~85	65~75	2800~3200

**Table 3:** Residual compressive strength and damage form of CF/EP composites under different impact energies at room temperature.

Specimen Number	Impact Energy/J	Residual Compressive Strength/MPa	Failure Mode
1	25	264.37	End failure
2	35	247.70	End failure
3	45	243.52	Valid LDM
4	65	222.16	Valid LDM
5	85	220.40	Valid LDM
6	105	191.14	Valid LDM
7	125	165.62	Valid LDM

## Data Availability

Data are available upon request due to restrictions on privacy. The data presented in this study are available on request from the corresponding author.
